# The unexpected always happens

**DOI:** 10.1186/2041-2223-3-5

**Published:** 2012-02-22

**Authors:** Mark A Jobling

**Affiliations:** 1 Department of Genetics, University of Leicester, University Road, Leicester LE1 7RH, UK

## 

Last October my son went off to University. Always a wrench, these life transitions, and they seem to happen so fast. One moment fighting in the bath with plastic dinosaurs, and the next moment leaving home, to do battle with the lecture, the launderette and the bank account. He has followed not in his father's, but in his mother's footsteps, choosing to study English Literature. His younger sister is also following a non-scientific career.

These days such subject choices, once made, seem irrevocable for students. Not so for one of my son's eminent predecessors, John Burdon Sanderson (J.B.S.) Haldane, who entered the same institution (New College, Oxford) exactly one hundred years before him. Destined to become one of the foremost evolutionary biologists, he began with a mathematics scholarship, and then switched to 'Greats' (Latin and ancient Greek), gaining a first-class degree under the looming shadow of the First World War [1]. Despite this circuitous path, his biological roots had been planted deep, in his relationship with his physiologist father. He first appears in print as last author of a paper in 1912, along with Haldane senior and C.G. Douglas [2], examining the uptake of oxygen and carbon monoxide by haemoglobin; J.B.S. did the maths, and here escaped being an experimental subject, the privilege of blood donation being reserved for the first two authors and a number of anonymous mice. Four years earlier he had already been experimenting in Mendelian genetics, breeding 300 guinea pigs on the lawn of their home with his sister Naomi. They eventually switched to the mouse, and published their study of linkage in 1915 [3].

In today's highly regulated world of research ethics and health & safety rules, where even ultrapure water purchased from Sigma carries a hazard warning, these early days seem as exciting as the wild west. Everything was there to be discovered, and self-experimentation was an excellent place to start. In working, with his father, to understand the effects of poison gases and to develop effective respirators, Haldane underwent gassing with chlorine. In experiments involving decompression chambers he suffered crushed vertebrae during a fit, and burst eardrums that, once healed with a hole remaining, left him somewhat deaf but permitted the social accomplishment of blowing tobacco smoke from the ears. In testing the effects of acidification of the blood he drank dilute hydrochloric acid, and sat in an airtight room containing 7% carbon dioxide, an exercise that 'gives one a rather violent headache'.

On preferring to experiment on himself than upon an animal, Haldane wrote that 'it is difficult to be sure how a rabbit feels at any time', and although dogs were better, '...to do the sort of things to a dog that one does to the average medical student requires a licence signed in triplicate by two archbishops' [4]. His dismissive attitude to the growing anti-vivisectionist movement came partly from his willingness to undergo pain himself in the pursuit of medical research (the animals, it must be said, did not have any option), and partly from his perception of the hypocrisy of some anti-vivisectionists, who included aristocrats keen on hunting.

Haldane's contributions to genetics were many and various. He formed part of the famous triumvirate, together with Sewall Wright and R.A. Fisher, who constructed the evolutionary synthesis, in which mathematical population genetics was used to reconcile Darwin's theory of natural selection with Mendel's rules of inheritance. He used quantitative methods, including maximum likelihood, to develop human linkage maps. He proposed that the high incidence of sickle cell anaemia was due to heterozygote advantage in malaria resistance [5]. He was also the first to estimate the human mutation rate, at 2 × 10^-5 ^mutations per gene per generation for the X-linked haemophilia gene [6]. This is equivalent to 2 × 10^-8 ^mutations per base per generation, assuming mutations at 1,000 bases could give rise to the disease, and remarkably close to the rate actually measured in pedigrees thanks to the industrial might of twenty-first century next-generation sequencing [7].

In a series of essays written for the public, Haldane popularised science; forthright, crystal-clear, and often very funny, his writing informs and educates while allowing the vivid character of this irascible polymath to shine through. Perhaps the most famous of the essays is *On being the right size *[8], which elegantly demonstrates the inevitable consequences that size brings for animal form - that 'a hare could not be as large as a hippopotamus, or a whale as small as a herring'. Haldane illustrates the problems that arise when linear size is increased ten-fold, but surface area a hundred-, and mass a thousand-fold. He imagines dropping different animals down a deep mine-shaft; while a mouse gets a slight shock and walks away, 'a rat is killed, a man is broken, a horse splashes'.

Haldane queried whether scientists should be rewarded for their work, and seemed to admire the French tradition of honouring researchers by naming streets and squares after them, rather than paying them. These days, scientists can get rewarded pretty well, but our achievements seem humdrum compared to his. What would Haldane work on, if he were around today? In one of his prescient essays [9], he speculates about gene manipulation ('if a hen's egg were the size of the world, we could get a gene into a room, and probably onto a small table'), and the synthetic cell. He also notes that 'animals have a chemical as well as a physical anatomy, and it will have to be taken into account in their classification', and it seems likely that comparative genomics and the new efforts to understand the molecular basis of development and its evolution would interest him. I would venture to suggest that a beautiful paper last year in *Nature *[10], identifying the deletion of a tissue-specific enhancer of the androgen receptor gene that is responsible for the happy fact that while chimpanzees have penile spines, men do not, would have tickled his fancy.

But there again, with Haldane, it's hard to know; as he himself said, the unexpected always happens. Maybe young Jobling will switch to Biology in a year two?
